# Risk factors for complications and adverse outcomes in polytrauma patients with associated upper extremity injuries

**DOI:** 10.1186/s13037-019-0187-3

**Published:** 2019-02-04

**Authors:** Florin Allemann, Sandro Heining, Boris Zelle, Christian Probst, Hans-Christoph Pape

**Affiliations:** 1 Department of Trauma, Universitaetsspital, University of Zurich, Raemistr.100, 8091 Zurich, Switzerland; 2 University of Texas Science Center at San Antonio, Floyd Curl Dr., 7703, San Antonio, TX 78229 USA; 3 Department of Trauma, Cologne-Merheim Med. Center, Ostmerheimerstr. 200, 51109 Koln, Germany

**Keywords:** Multiply injured patient, Upper extremity fracture, Articular fracture, Heterotopic ossification

## Abstract

**Background:**

In terms of upper extremity fractures by patients with multiple injuires, a lot of studies have assessed the functional outcome following trauma to have less favorable outcomes in regards to functional recovery. We tested the hypothesis that differences in clinical outcome occur between shaft and articular injuries of the upper extremity, when patients that sustained neurologic deficits (e.g. brachial plexus lesions) are excluded.

**Methods:**

We involved Patients with isolated or combined upper extremity fracture, ISS > 16 in a level one trauma center. The follow up was at least 10 years after the initial injury. Both clinical examination (range of motion, instability, contractures, peripheral nerve damage) and radiographic analysis were carried out. We evaluated also the development of heterotopic ossifications. To analyse patients were subdivided into 3 different subgroups (articular [IA], shaft [IS], and combined [C]).

**Results:**

A statistically significant difference was found when ROM was compared between Group IS and C (*p* = 0.012), for contractures between Groups IA and C (*p* = 0.009) and full muscle elbow forces between Groups IS and C (*p* = 0.005) and Group IA and IS (*p* = 0.021). There was a significantly increased incidence in heterotopic ossifications when articular involvement was present. This applied for the isolated (*p* < 0.02) and the combined group (Group C vs Group IS, *p* = 0.003).

When Brooker type I/II in group IA and Brooker types III/IV were combined, there was a significant difference (*p* < 0.001). In group IA (*n* = 1) and in group C (*n* = 6), HO developed or worsened after revision surgery, all of which were performed for malunion or nonunion.

**Conclusions:**

In this study, patients with isolated shaft fractures of the upper extremity tend to have a more favorable outcome in comparison with combined to isolated articular fractures in terms of range of motion, pain and the ability to use the arm for everyday activities.

In the clinical practice of the treatment of polytraumatized patients with upper extremity injuries, we feel that the relevance of these injuries should not be underestimated. They are especially prone to development of heterotopic ossifications, thus requiring prophylactic measures, if necessary. As their incidence increases with the rate of reoperations, we feel that even during initial care, meticulous surgery is required to avoiding the necessity of revision surgeries. Similar to injuries below the knee, upper extremity injuries, should be treated to avoid any functional disability.

## Background

In orthopaedic trauma patients, it has been one of the main goals to re-establish functionality that allow the patient to return to activities of daily life (ADL). In those patients that sustained multiple injuries, functional recovery has been a special area of focus along with recent improvements in survival rates [1–3]. This is known to be a long-term process and multiple studies have investigated outcome with regards to upper and lower extremity injuries [4–16]. Zelle et al. [16] demonstrated that fractures below the knee joint strongly influenced the functional recovery following multiple injuries. Unfavorable outcomes were associated with delayed treatment, a thin soft tissue envelope, high energy trauma, unfavorable blood supply, and complex fracture patterns.

In terms of upper extremity fractures, multiple studies have assessed the functional outcome following trauma [17–27], of which especially fractures in the elderly population tend to have less favorable outcomes in regards to functional recovery [28–31]. In addition, other factors, such as female gender, high injury severity, head injuries, insurance by workers’ compensation and lower educational level have been identified to be linked to poor outcome [4–6, 9, 10, 32–34]. To our knowledge, almost available studies on follow up of the upper extremity have focused on isolated injuries. Dysfunction assessment after upper extremity fractures has frequently focused on issues of impaired range of motion [18, 19, 26, 28], non-union [24, 35–37], and heterotopic ossification [33, 38, 39].

Our group has previously focused on a comparison of multiply injured patients with and without injuries to the upper extremity and found limited to no effect of the additional upper extremity trauma. The only relevant cofactor for impaired outcome was the presence of a brachial plexus injury [3].

In terms of local complications in polytrauma patients, articular injuries appear to be particularly sensitive to the development of heterotopic ossifications, more than shaft fractures [4]. We therefore tested the hypothesis that differences in clinical outcome occur between shaft and articular injuries of the upper extremity, when patients that sustained neurologic deficits (e.g. brachial plexus lesions) are excluded.

## Methods

The study was approved by the local Institutional Review Board, as addressed previously approved the study protocol and all participants provided written informed consent [40].

### Study population

**Fig. 1:**
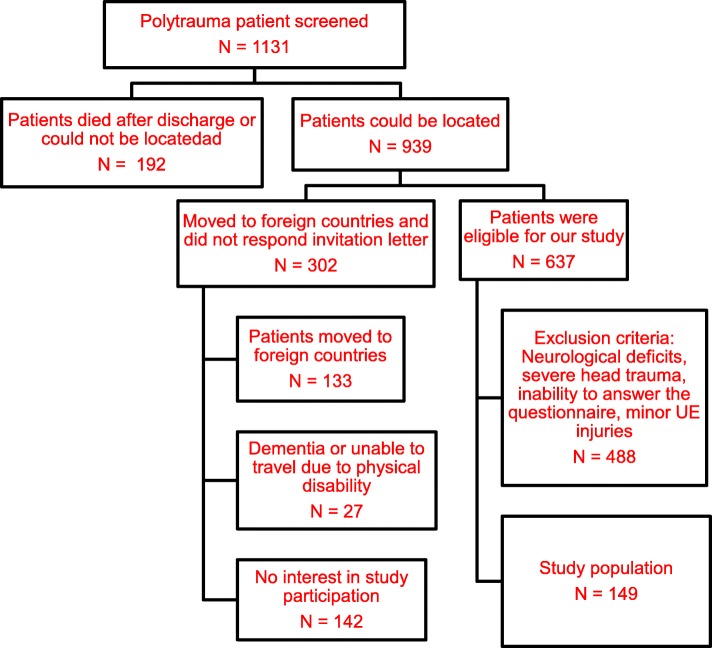
Flowchart

The recruitment process has been described in detail in a previous publication. In this investigation patients were reexamined at a minimum of 10 years after injury [40]. All patients were contacted by conventional surface mail. Patients were asked to contact the phone number provided on the letter and asked to make a follow up appointment. If patients did not reply to the first letter, follow up letters were sent by surface mail, up to three reminders. Patients were called at home to schedule an appointment if no response had been obtained after the third letter. The local government office for registration of residents was contacted if the patient had moved to obtain the patient’s new address (Fig. 1).

### Clinical examination

The physician involved in the follow-up analysis also performed all follow-up examinations. The results of these routine examinations are summarized in reporting letters. This concept has been selected to increase the likelihood of detecting minor lesions during the clinical course in the normal ward, which may have been overlooked at the time of admission.

### Inclusion and exclusion criteria

*Inclusion criteria “upper extremity study”*

- ➢Patients with isolated articular or shaft fractures, isolated upper extremity fracture.
- ➢Patients with combined (two or more) upper extremity fractures.
- ➢Multiple injuries (ISS greater or equal 16 points)
- ➢Treatment at one level one trauma center
- ➢age between 3 and 60 years at the time of injury
- ➢Follow up examination at least 10 years after the initial injury

*Exclusion criteria:*

- ➢Patients who sustained neurologic deficits, e.g. brachial plexus injuries [3]
- ➢Patients who had moved to more than three different communities or a foreign country since their injury
- ➢Patients who did not register their new address at the local government office for registration of residents
- ➢Patients who declined participation in this study and/or had missed more than three follow up appointments
- ➢Patients who did not respond to three consecutive recruitment letter and did not respond to several consecutive phone calls.

We have evaluated the Injury severity by using the Injury Severity Score (ISS) [41], calculated based on the Abbreviated Injury Score (AIS, Version 1990) for every body region. Additionally, Heterotopic ossifications (HO) were assessed as previously described [4] and quantified by using the grading system of Brooker et al. [33].

The Neutral-0-method was used in unilateral and bilateral injuries of wrist, elbow and shoulder to determine Range of Motion (ROM). Three different ranges were separated, in comparison with the normal range of a population of healthy volunteers.

The 12-Item-Short-Form Health Survey (SF-12) [42] was used for both, the psychological and physical outcome by gathering information in a questionnaire.

A published rehabilitation score [43] was used for the evaluation of subjective and objective findings in outcome. As described previously [40], all patients were assessed by a physician, using a standardized self-administered patient questionnaire and a standardized physical examination of the injured body regions.

It consists of two parts. Part 1 is filled out by the patient and deals with quality of life for the patient in general and social-economical issues. Part 2 focuses on findings of the physical exam for every body region and the rehabilitation status.

The general outcome measurements included upper extremity specific outcome measurements and general outcome measurements: range of motion, weight bearing status of the injured lower extremity, persistent pain, and function.

For the shoulder, the ROM of every joint was examined for flexion and extension, internal and external rotation both at 90 degrees and in resting position, and abduction and adduction. For the elbow we examined flexion and extension and pronation and supination. For the wrist ROM was examined for flexion and extension and ulnar and radial abduction.

Patients were subdivided into 3 different subgroups as follows.

*Group IA: Isolated Articular* fractures (shoulder fractures, i.e. humeral head and scapular fractures; elbow fractures, i.e. supra−/epicondylar humeral fractures, proximal ulna and radius fractures; wrist fractures, i.e. distal radius and ulna fractures).

*Group IS: Isolated Shaft* fractures (humeral shaft, radius shaft, ulnar shaft fractures).

*Group C: Combined fractures* (shaft and articular, shaft and shaft, or articular and articular fractures).

### Radiographic analysis and development of heterotopic ossifications

In all patients with upper extremity injuries, healing of the fracture is routinely assessed by radiographic control at 6 and 12 weeks, or until osseous healing had occurred. In patients that underwent revision surgeries, their course was also followed clinically and radiographically until final fracture healing.

### Outcome parameters

The following parameters were assessed during the orthopedic exam:

- ➢ROM greater than 50 degree in the affected joint
- ➢Instability in the affected joint by clinical examination
- ➢Stiffened joints, contractures, blood circulation disorders, weakness and/or peripheral nerve damage
- ➢Number of patients with full wrist, elbow and shoulder muscle force (compared with the uninjured side). The muscle force was graded on a clinical scale from 0 to 5, as used in neurological examinations.

The following items were covered by the standardized patient questionnaires

- ➢Ability to use the upper extremity at work/hobby/sports related activities
- ➢Ability to carry out complex movements
- ➢pain in affected region
- ➢full improvement during the healing process

### Statistics

Age at the time of injury was divided into quartiles (3–18; 19—23; 24—33 and 34—60 years); the ISS was divided into three groups (0—15; 16—25; > 25); the mental and physical component summary scores of the SF-12 were divided into four groups (0—30; 31—40; 41—50; > 50); the rehabilitation score values were divided into quartiles (< 27.9; 27.9—54.4; 54.4—89.5; > 89.5).

Nonparametric tests were used as follows: for group comparisons, the Kruskal-Wallis Test was used, if applicable. Continuous variables were compared using the Mann Whitney U test. *P*-values of less than 0.05 were considered significant. The tables list median and quartiles (P25-P75), if needed.

## Results

Of the 1.131 patients eligible (76% men), 103 patients (83% men) died after discharge and 89 (73% men) patients could not be located. 939 (76% men) of the eligible patients were found to be alive at follow-up and could be successfully located, and thus were potential enrollees for this study. Among the 637 patients that were seen for interview and physical examination, 149 patients had major injuries of the upper extremity and were included in the current study. Table 1 demonstrates the demographic data of all patients included in this study.

**Table 1:** Demographic data of all patients with upper extremity injuries

Number of patients	149
Gender	74.5% M/ 25.5% F
Age at accident (years)	
3-18	16 ± 5.9
19-23	23 ± 5.0
24-33	29 ± 4.9
34-60	55 ± 7.1
Age at follow up (years)	44 ± 11.9
ISS	
0-15	5 ± 4.1
16-25	16 ± 6.5
> 25	26 ± 4.8
Head Concussion (light head injury n, %)	89 (59.7%)
Head Contusion (mod. Head injury n, %)	5 (0.3%)
Time between accident and F/U (years)	17.1 yrs. ± 5
Patients that underwent surgery n (%)	88 (59.1%)
ICU stay (days)	13.6 days ±22.4
Multiple upper extremity fractures	52 (34.9%)
Isolated upper extremity fractures	60 (40.3%)
Upper extremity shaft fractures	37 (24.8%)
Sf 12 (psychological)	50.9 ± 10.5
Sf 12 (physical)	44.4 ± 10.8
Rehab score (HASPOC)	64.1 ± 44.9

Sixty patients belonged to Group IA, 37 patients in Group IS and 52 patients belonged to Group C. Table 2 shows the demographic data and AIS scores of all three groups. There was no difference in the incidence of patients for AIS subgroups I-III. There were no differences in demographics of the subgroups and they were thus not mentioned. A statistically significant difference was found when the standardized Rehabilitation score was compared in certain subgroups, and for the SF-12 physical between Group IS and C (Table 2).

**Table 2:** General assessment of outcome in scoring systems

Patient Group	Group IA	Group C	Group IS	*p*-value
# of patients	60	52	37	n.s.
ISS	21.7 ± 5.9	25.7 ± 5.1	22.2 ± 6.6	n.s.
AIS I (n, %)	25 (41.6)	21 (40.4)	12 (32.4)	n.s.
AIS II (n, %)	27 (45.0)	25 (48.1)	14 (37.8)	n.s.
AIS III (n, %)	8 (13.4)	6 (11.5)	11 (29.7)	n.s.
SF-12 psychological				
0 – 30	21.7 ± 5.2	22.6 ± 7.6	23.3 ± 4.1	n.s.
31 – 40	35.6 ± 3.1	37.5 ± 4.9	38.2 ± 6.1	n.s.
41 – 50	45.6 ± 4.9	47.3 ± 2.9	48.0 ± 6.2	n.s.
> 50	53.3 ± 9.2	55 ± 6.9	59 ± 5.9	n.s.
SF-12 physical				
0 – 30	24.2 ± 2.6	27.3 ± 4.8	21.3 ± 3.1	*p* = 0.03^§^
31 – 40	36.2 ± 4.3	38.4 ± 4.3	39.3 ± 8.1	n.s.
41 – 50	44.3 ± 5.1	45.3 ± 5.1	49.7 ± 3.8	*p* = 0.04^§^
> 50	50.3 ± 4.9	51 ± 4.3	55.1 ± 9.3	n.s.
Rehabilitation score				
< 27.9	15.3 ± 6.2	17.3 ± 5.8	14.2 ± 3.9	n.s.
27.9—54.4	37.3 ± 5.3	46.3 ± 3.8	29.9 ± 2.4	*p* = 0.044^§^
54.4—89.5	75.2 ± 5,9	73 ± 3.9	66 ± 6.9	*P* < 0.47^#^
> 89.5	91.0 ± 3.5	95.1 ± 4.1	90.9 ± 4.9	n.s.

(#) Comparison between combined and shaft fractures

(+) Comparison between isolated articular and combined fractures

(§) Comparison between isolated articular and shaft fractures and group C

Table 3 documents the findings during the physical exam in all patient subgroups.

**Table 3:** Findings of the physical exam in isolated articular, combined and shaft fractures

Patient Group	Group IA	Group C	Group IS	*p*-values
# of patients	60	52	37	n.s.
ROM > 50% (n)	53 (88.3%)	38 (73.1%)	35 (94.6%)	*P* = 0.012^#^
ROM 20 – 50% (n)	2 (3.33%)	5 (9.6%)	2 (5.4%)	n.s.
ROM < 20%(n)	6 (10%)	9 (17.3%)	0 (0%)	n.s.
Instability elbow (n)	0 (0%)	1 (1.9%)	1 (2.7%)	n.s.
Contracture (n)	5 (8.33%)	13 (25%)	4 (10.8%)	*P* = 0.009^+^
Heterotopic ossification	22 (36%)	31 (59%)	5 (13%)	*P* = 0.003^#^ *P* < 0.02^§^
AR	0 (0%)	4 (7.7%)	1 (2.7%)	n.s.
Neurological impairment (n)	7 (11.7%)	7 (13.5%)	4 (10.8%)	n.s.
Full muscle force: shoulder	54 (90%)	46 (88.5%)	36(97.3%)	n.s.
Full muscle force: elbow (n)	52 (86.7%)	42 (80.8%)	37 (100%)	*P* = 0.021^§^ *P* = 0.005^#^

(#) Comparison between combined and shaft fractures

(+) Comparison between isolated articular and combined fractures

(§) Comparison between isolated articular and shaft fractures

A statistically significant difference was found when ROM was compared between Group IS and C (*p* = 0.012), for contractures between Groups IA and C (*p* = 0.009) and full muscle elbow forces between Groups IS and C (*p* = 0.005) and Group IA and IS (*p* = 0.021). There was a significantly increased incidence in heterotopic ossifications when articular involvement was present. This applied for the isolated (*p* < 0.02) and the combined group (Group C vs Group IS, *p* = 0.003).

Table 4 depicts the subgroup analysis for patients that developed heterotopic ossifications. Patients with isolated shaft fractures (Group IS) demonstrated the lowest incidence of HO overall, all of them were low degree (Brooker type I). In Group IA, the majority of patients developed grade I and II lesions, whereas the in group C most patients developed HO’s of a larger degree (Brooker III and IV). When Brooker type I/II in group IA and Brooker types III/IV were combined, there was a significant difference (*p* < 0.001). In group IA (*n* = 1) and in group C (*n* = 6), HO developed or worsened after revision surgery, all of which were performed for malunion or nonunion.

**Table 4:** Subgroup analysis of patients with heterotopic ossifications

Patient Group	Group IA	Group C	Group IS	*p*-values
# of patients	22	31	5	
Brooker grade I	**10 (45.5%)**	1 (3.2%)	5 (100%)	*P* = 0.006^#^ ns^+^ *p* < 0.01^§^ *P* < 0.001 (I/II vs III/IV)
Brooker grade II	**9 (40.9 (%)**	5 (16.12%)	–	*P* < 0.002^#^ *P* < 0.001^+^ n.s.^§^
Brooker grade III	1 (4.6%)	**15 (48.4%)**	–	*P* < 0.002^#^ *P* < 0.001^+^ n.s. (§)
Brooker grade IV	2 (0.1%)	**10 (32.3%)**	–	
HO < 1 year post trauma	19	24	5	*P* = <.002^#^ ns^+^ *p* < 0.01^§^
HO > 1 year post trauma	2	1	–	n.s.
HO after revision surgery	1	6	–	n.s.

(#) Comparison between combined (group C) and shaft fractures (group IS)

(+) Comparison between isolated articular (group IA) and combined articular and shaft fractures (group C)

(§) Comparison between isolated articular (IA) and shaft fractures (IS)

## Discussion

Trauma of the extremities continues to represent a substantial health care problem in civilized countries and causes a substantial burden on society and cost. Among the studies focusing on outcome, those in patients with multiple injuries have gained increasing importance and most of them focused on lower extremity injuries. Certain subsets of injuries, such as injuries below the knee, or those with neurologic deficits associated with spinal cord injuries are known to be less successful in terms of recovery [44–47].

In a previous study, we have evaluated general risk factors on the impact on clinical outcome using a binary logistic regression analyses. In that particular study, we have found that the only predictors in of poor outcome were traumatic lower extremity amputation and a high initial maximum AIS spine score, indicating initial para- or tetriplegia.

The outcome after upper extremity fractures has been studies in multiple clinical examinations. Most of these reports focused on certain fracture types and were followed for a shorter period of time. The main focus was usually laid on outcome issues following a certain fixation technique, or other orthopaedic issues [18, 19, 21–23, 26, 27, 48]. The only previous long term work up was published from our group and compared patients with and without upper extremities. It demonstrated that the additional upper extremity lesion itself does not have a sustained effect on outcome, unless there is an associated plexus lesion [3]. However, local injury patterns may have a sustained effect on the complication rate, and can be induced by heterotopic ossifications, ipsilateral injuries and other factors [4, 23, 26, 27].

We feel that our current study has both strengths and limitations. The long follow-up period and the well defined patient group selection may be regarded as a strength. Moreover, all examinations were performed by a physician. Also, once the diagnosis of heterotopic ossification had been made, we were able to track for the timing of it, along with influences of follow-up surgeries, as outlined below.

### Drawbacks of the study

Although a long term follow up has not been available so far, we are aware that the long range of follow up (10 to 17 years) might represent a downside. Rehabilitation protocols may have changed and therefore could have influenced the results obtained. Thus, an affect on the range of motion may have been an issue. Moreover, we have been unable to exclude any effects of additional injuries on the outcome of the upper extremity. Finally, the exclusion of patients with head injury could have had an impact. As head injured patients are known to be at special risk for the development of hetertotopic ossifications, the exclusion of this subgroup might have promoted false positive values of the range of motion.

Therefore the impact of the upper extremity lesion may be of limited value for the general score results of SF 12 and others.

We tried to account for this by using a focused clinical examination as part of the initial assessment. Thereby, we were able to differentiate changes in the range of motion, neurologic deficits, strength assessment and other local problems.

Given these prerequisites, our main results were as follows:

1. Patients with isolated shaft fractures (Group IS) of the upper extremity had the best outcome in full recovery in comparison with combined and isolated articular fractures. This applied with respect to ROM, pain, neurological impairment and the ability to use the extremity for work and sports-related activities. Similar results had also been described in a study by Ekholm et al. [49]. In their stuy, 25–30% of patients with isolated articular fractures and combined fractures did not show full recovery when the short musculoskeletal functional assessment test was used. Therefore, our subanalysis provides more subtle information regarding the outcome when special focus was laid on the differentiation of shaft versus articular fractures. We feel that this information is helpful in the overall assessment of the patient with multiple injuries and provides additional information to that reported previously in a different patient subset (4). These results included arm/hand function, daily activities, emotional status and mobility, at follow up.
2. Patients with isolated articular fractures regained full elbow muscle force in as low as 87%, when compared with 81% in combined fractures and 100% of shaft fractures.

This may be explainable by the fact that patients with combined fractures could have had fractures in two regions close to or within the elbow. To our knowledge only one study [50] describes follow up in musculoskeletal injuries 5 years after accident and concluded that the majority of patients had not gained full recovery [51]. This could be due to the fact that only a small percentage of upper injuries result from high-energy trauma usually leading to a severe injury pattern [52].

3. In patients with articular involvement of an upper extremity (Grous IA and C) demonstrated a sustained increase in the risk of heterotopic ossifications (HO), when compared with isolated shaft fractures

These results are in line with previous studies from our group that demonstrate an increased rate of HO depending on issues of local soft tissue pressures, and other factors unrelated to the initial injury [4]. Also these results are not surprising, as HO prophylaxis in general has been recommended in certain scenarios, especially for revision surgeries around the elbow. In this line, it was interesting to recognize that sustained differences in outcome occured in the subgroup analysis with heterotopic ossifications. The majority of patients with HO in Group C developed Brooker III and IV degrees of HO, whereas Group IA usually developed Brooker I and II lesions. When taken together, this trend was significant despite the low patient number. Finally, it was interesting to observe that in both groups, HO developed after surgery when there was an articular injury.

These results are in line with our finding that patients with combined fractures of the upper extremity also demonstrated a worse score result than patients with isolated fractures. A normal ROM was found in 88% of patients with isolated articular fractures and in 73% of patients with combined fractures. Similar trends had been described by Stalp et al. [43] in a 2 year follow up study.

In our study, patients from all three groups showed the same distribution of arterial injuries of the upper extremity and no specific pattern of arterial or neurological injuries. In a similar fashion, a study by Joshi et al. [53] dealt with the overall outcome of patients with blunt and penetrating trauma to the upper extremity with respect of neurological and arterial impairment over a 5-year period and they concurred with our study that patients with blunt trauma are more prone to neurological than arterial injuries. One limitation to their study is the relatively small study population. They also came to the conclusion that patients with multiple injuries involving the upper extremity show longer initial hospitalization and increased disability following multi-trauma in a period. In comparison to their study our study shows that patients have a fairly good long-term outcome regarding their upper extremity injury.

## Conclusions

We conclude patients with isolated shaft fractures of the upper extremity tend to have a more favorable outcome in comparison with combined to isolated articular fractures in terms of range of motion, pain and the ability to use the arm for everyday activities. This might be explained by the fact that the shaft is less prone to continuous movement than the joint. Also, the patients with articular involvement appeared to be particularly susceptible to the development of heterotopic ossifications and requirement of revision surgeries. Therefore, special focus in terms of surgical treatment and rehabilitation should be applied for patients with combined upper extremity injuries.

In the clinical practice of the treatment of polytraumatized patients with upper extremity injuries, we feel that the relevance of these injuries should not be underestimated. They are especially prone to development of heterotopic ossifications, thus requiring prophylactic measures, if necessary. As their incidence increases with the rate of reoperations, we feel that even during initial care, meticulous surgery is required to avoiding the necessity of revision surgeries. Similar to injuries below the knee, upper extremity injuries, should be treated to avoid any functional disability.

## Abbreviations

AIS (Version 1990): Abbreviated Injury Score
HO: Heterotopic ossification
ISS: Injury Severity Score
ROM: Range of Motion
SF-12: 12-Item-Short-Form Health Survey
